# Stronger warming effects on microbial abundances in colder regions

**DOI:** 10.1038/srep18032

**Published:** 2015-12-10

**Authors:** Ji Chen, Yiqi Luo, Jianyang Xia, Lifen Jiang, Xuhui Zhou, Meng Lu, Junyi Liang, Zheng Shi, Shelby Shelton, Junji Cao

**Affiliations:** 1 State Key Laboratory of Loess and Quaternary Geology (SKLLQG), and Key Laboratory of Aerosol Chemistry and Physics, Institute of Earth Environment, Chinese Academy of Sciences, Xi’an 710061, China; 2 University of Chinese Academy of Sciences, Beijing, 100049, China; 3 University of Oklahoma, Department of Microbiology and Plant Biology, Norman, 73019, USA; 4 Tsinghua University, Center for Earth System Science, Beijing, 100084, China; 5 Fudan University, Coastal Ecosystems Research Station of Yangtze River Estuary, Ministry of Education Key Laboratory for Biodiversity Science and Ecological Engineering, The Institute of Biodiversity Science, Shanghai 200433, China,; 6 Xi’an Jiaotong University, Institute of Global Environmental Change, Xi’an 710049, China

## Abstract

Soil microbes play critical roles in regulating terrestrial carbon (C) cycle and its feedback to climate change. However, it is still unclear how the soil microbial community and abundance respond to future climate change scenarios. In this meta-analysis, we synthesized the responses of microbial community and abundance to experimental warming from 64 published field studies. Our results showed that warming significantly increased soil microbial abundance by 7.6% on average. When grouped by vegetation or soil types, tundras and histosols had the strongest microbial responses to warming with increased microbial, fungal, and bacterial abundances by 15.0%, 9.5% and 37.0% in tundra, and 16.5%, 13.2% and 13.3% in histosols, respectively. We found significant negative relationships of the response ratios of microbial, fungal and bacterial abundances with the mean annual temperature, indicating that warming had stronger effects in colder than warmer regions. Moreover, the response ratios of microbial abundance to warming were positively correlated with those of soil respiration. Our findings therefore indicate that the large quantities of C stored in colder regions are likely to be more vulnerable to climate warming than the soil C stored in other warmer regions.

An important ongoing endeavor in current global change ecology and biology is incorporating microbial responses into Earth system models (ESMs), as microbes have been highlighted as one of main unknowns controlling the fate and turnover of soil organic matter (SOM)^1^,^2^,^3^. Modeling studies predicted that climate warming would stimulate microbial decomposition of SOM, representing an important positive feedback loop^4^,^5^. Although short-term field experiment documented an initial increase of soil respiration (SR) to climate warming^6^, there were still large uncertainties in the strength and magnitude of the effects of warming on ecosystem carbon (C) cycles^7^,^8^. Such large uncertainties might be mainly due to the response of the soil microbial activities^1^, since warming-induced changes in the soil microbial abundance had the potential to either accentuate^9^,^10^ or mitigate^7^,^11^ warming-induced C losses substantially. However, it remains unclear, especially in broad-scales evaluation of how microbial abundance respond to global climate change.

The effects of warming on soil microorganisms and the causal mechanisms linking the two are varied with climate regions and ecosystem types. Firstly, warming significantly enhanced microbial abundance in alpine regions and subarctic regions^12^,^13^, whereas the effects of warming on microbial abundance in temperate regions were often neutral or negative^11^,^14^. Possible explanations were associated with the microbial temperature sensitivity and microbial adaptation strategies^15^, although these explanations were still highly disputed^16^. On the other hand, warming often decreased microbial abundance in the temperate forest^17^,^18^, while enhanced it in the tundra or peat-land^12^,^13^,^14^. These inconsistent responses were mainly attributed to the soil substrate availabilities and soil properties^19^,^20^. These types of divergent responses greatly hampered our understanding of how microbial abundance responds to warming and what are underlined mechanisms. Therefore, a synthesis that aims to examine the broad-scale responses of microbial abundance to warming among ecosystem types and climate regions is necessary.

Two recent meta-analyses concluded that changes in microbial abundance were significantly correlated with changes in SR following ecosystem disturbances or N additions^21^,^22^. However, to our knowledge, it remains unclear whether there are some links between the responses of microbial abundance and SR to warming in large scales. To advance the predictive ability in regard to microbial abundance and associated SR under warming scenarios, we conducted a meta-analysis on the responses of microbial abundance to warming. First, we hypothesized that warming could have positive effects on microbial abundance. Second, we hypothesized that the responses of microbial abundance to warming would vary with climate regions and ecosystem types. Finally, we hypothesized that the responses of microbial abundance could be tightly coupled with changes in SR. We also tested above hypotheses separately for fungi, bacteria and Archaea.

## Results

### Microbes

**Figure 1:**
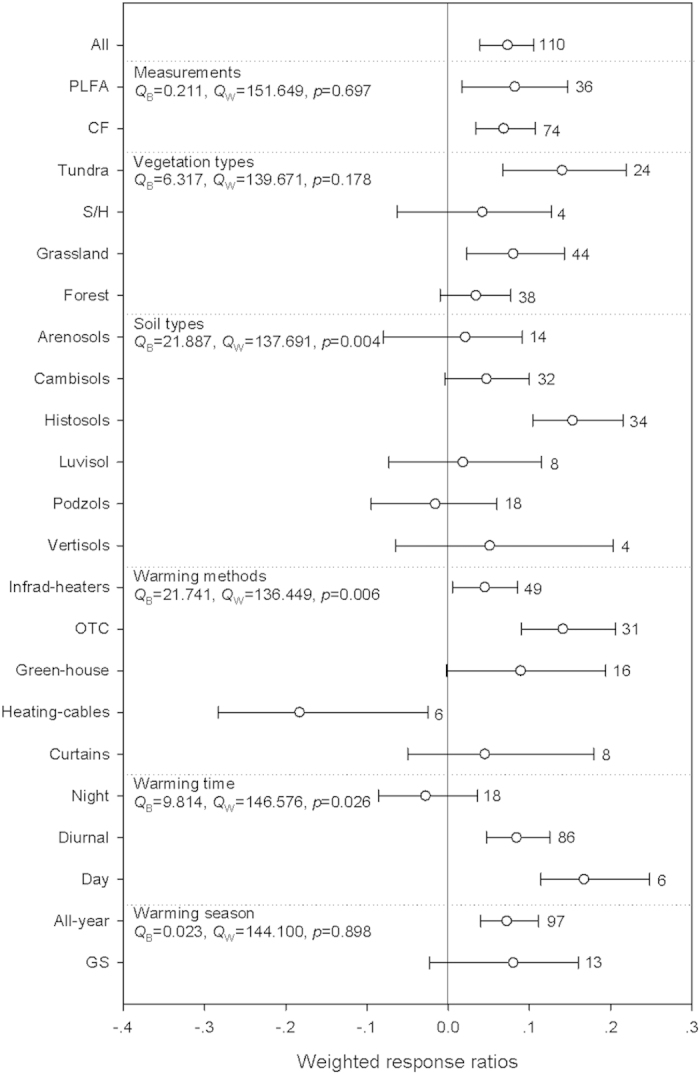
Warming effects on microbial abundance. Error bars represented bootstrap 95% confidence intervals (CIs). The effect of warming was considered significant if the CI of the effect size did not cover zero. The sample size for each variable was shown next to the CI. The definition of Q_B_ and Q_w_ can be found in materials and methods section, the *p* values indicate the difference within groups (*Q*_*w*_). The vertical solid line was drawn at mean effect size = 0. CF: chloroform fumigation, S/H: shrubland/heathland, GS: growing season, NGS: non-growing season. More information about the the percentage change for each variable and the weighted responses ratios in different warming magnitude for each variable can be found in supplemetary Table 1.

Across all studies, soil microbial abundances significantly increased following warming by an average of 7.6%, and there was no publication bias (Fig. 1, Table 1, Supplementary Table 1). When grouped by measurements, warming significantly enhanced microbial abundance by 8.5% and 7.0% as measured by total amount of phospholipid fatty acids (PLFA) and chloroform fumigation (CF), respectively. Within vegetation types, warming significantly increased microbial abundance in tundras by 15.0% and grasslands by 8.3%. Regarding to soil types, warming significantly increased microbial abundance in histosols by 16.5%. With reference to warming methods, warming by infrared-heaters and open top chambers (OTC) significantly increased microbial abundance by 4.6% and 15.1%, respectively, while warming by heating-cables decreased it by 16.7%. When grouped by warming time, diurnal and day warming significantly enhanced microbial abundances by 8.7% and 18.1%, respectively. In assessment of warming season, all-year warming treatments significantly enhanced microbial abundances by 7.5%. Significant between-groups heterogeneities were found when grouped by soil types, warming methods, and warming time.

There was significant negative relationship between the response ration (RR) of microbial abundance and warming magnitude. But, there was no relationship between the RR of microbial abundance and warming duration, mean annual precipitation (MAP), latitude or elevation, even when grouped by different warming magnitudes (Supplementary Figs 1–3). In addition, warming also significantly increased the abundance of Actinomycetes, Saprotrophic fungi, and the ratio of Bacteria: Fungi (Table 1).

### Fungi

**Figure 2:**
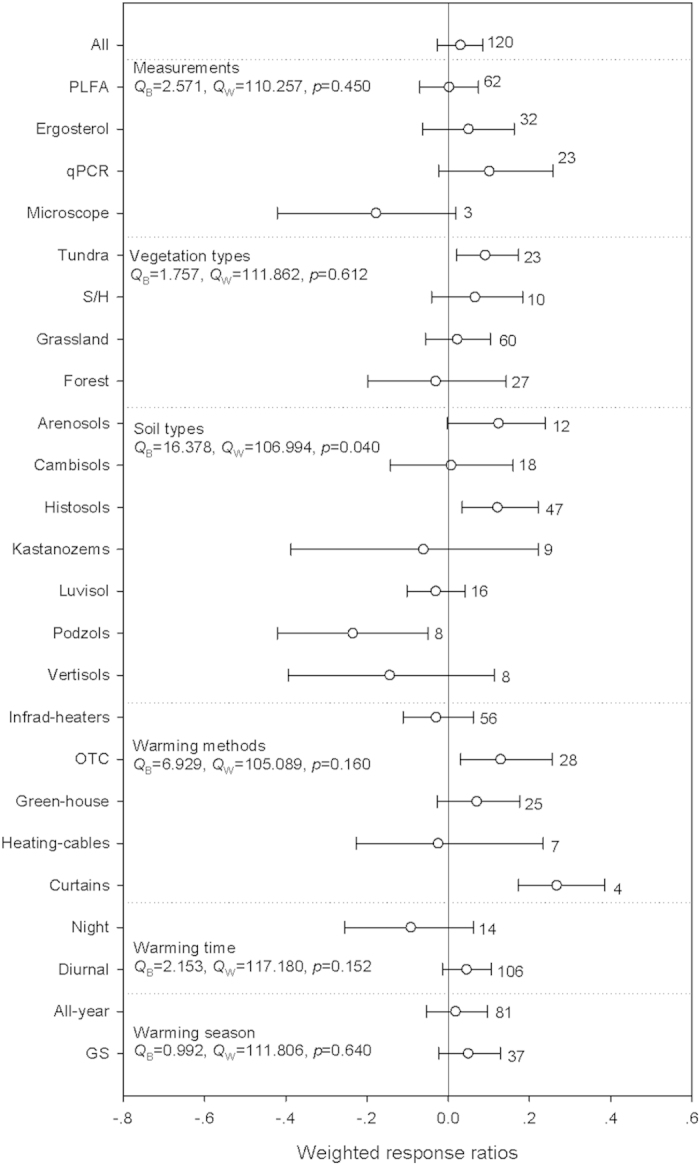
Warming effects on fungal abundance. See Fig. 1 for detailed information. More information about the the percentage change for each variable and the weighted responses ratios in different warming magnitude for each variable can be found in supplemetary table 2.

Fungal abundances did not significantly respond to warming (Fig. 2, Supplementary Table 2). Regarding to vegetation types, warming significantly increased fungal abundances in Tundras by 9.5%. Within soil types, warming significantly increased fungal abundance in histosols by 13.2%, while warning decreased it in podzols by 20.9%. With respect to warming methods, warming by OTC and curtains significantly increased fungal abundances by 13.8% and 30.7%, respectively. The between-groups heterogeneity was not significant excepted when grouped by soil types (*p* = 0.040).

There was a significant negative relationship between the RR of fungal abundance and warming magnitude and MAP. The relationship for MAP did not hold true when the results from the highest MAP site were excluded, but this relationship for MAP still hold true under medium warming magnitude. In addition, we did not find a significant relationship between the RR of fungal abundance and warming duration, latitude and elevation, even when grouped by different warming magnitudes (Supplementary Figs. 1–3).

### Bacteria

**Figure 3:**
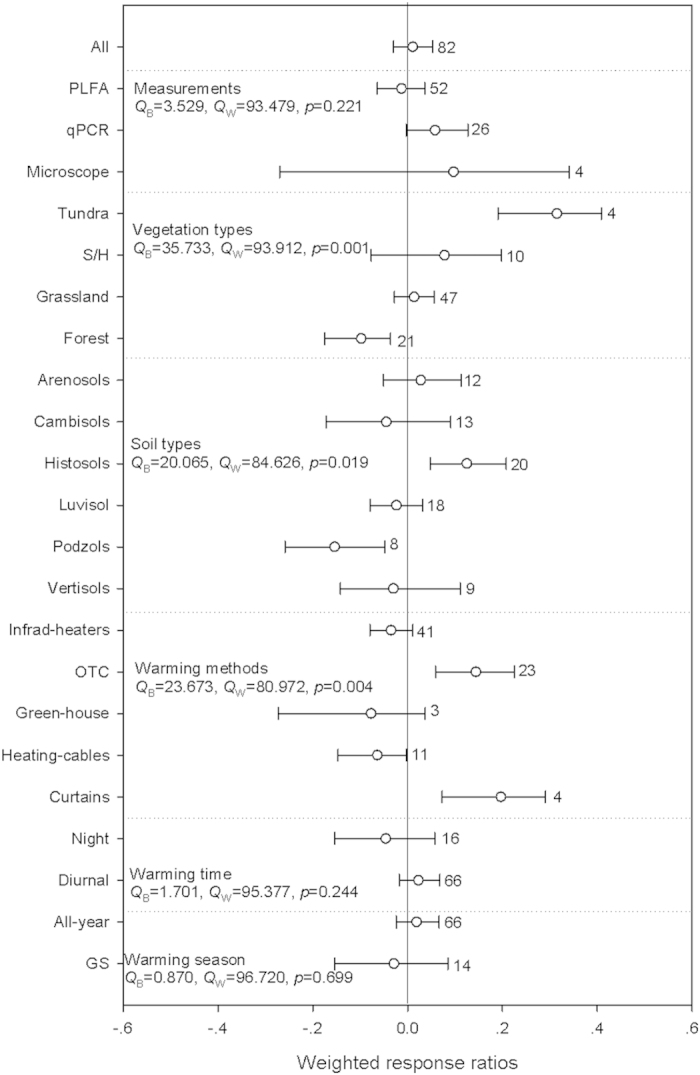
Warming effects on bacterial abundance. See Fig. 1 for detailed information. More information about the the percentage change for each variable and the weighted responses ratios in different warming magnitude for each variable can be found in supplemetary table 3.

Warming had no effect on overall bacterial abundance (Fig. 3, Supplementary Table 3), and no significant between-groups heterogeneity was found for the various measurement methods. The response of bacterial abundance differed among the vegetation types (*p* = 0.001), warming significantly increased bacterial abundance in tundras by 37.0%, but warming decreased it in forests by 9.3%. Regarding to vegetation types, warming significantly increased bacterial abundance in histosols by 13.3%, but decreased it in podzols by 14.3%, and there was significant between-groups heterogeneity for soil types. With reference to warming methods (*p* = 0.004), warming by OTC and curtains, significantly increased bacterial abundance by 15.5% and 21.8%, respectively, while warming by heating-cables significantly decreased it by 6.2%.

There was a significant negative relationship between the RR of bacterial abundance and warming magnitudes. But, there was no relationship between the RR of bacterial abundance and warming duration, MAP, latitude or elevation, even when grouped by different warming magnitudes. When these analysis were grouped by warming magnitudes, significant relationship was only found for MAP under low warming magnitude (Supplementary Figs 1–3).

### Archaea

**Figure 4:**
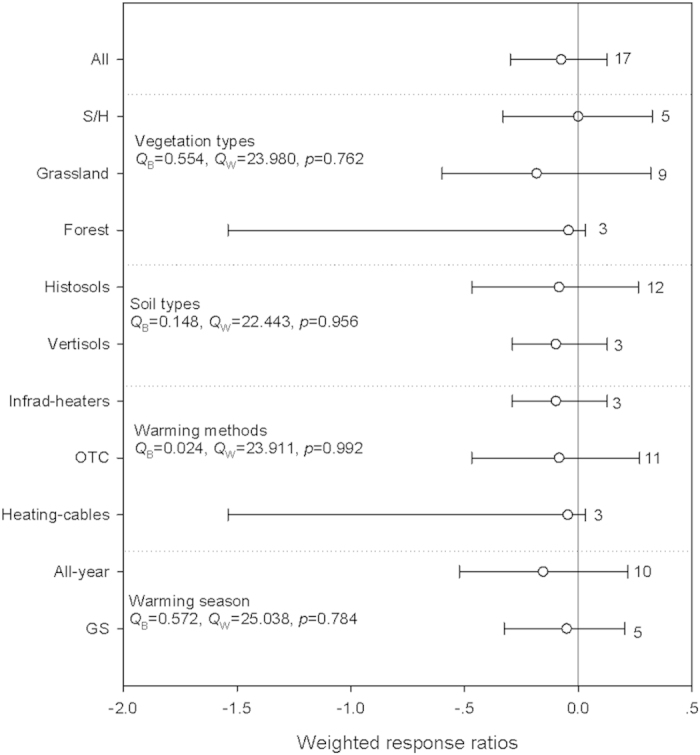
Warming effects on Archaea abundance. See Fig. 1 for detailed information. More information about the the percentage change for each variable and the weighted responses ratios in different warming magnitude for each variable can be found in supplemetary table 4.

Warming had no significant effect on Archaeal abundance (Fig. 4, Supplementary Table 4). We did not detect any significant difference between-groups heterogeneities in the analyses of Archaea abundance. In addition, there were no significant relationships between the RR of Achaea abundance and mean annual temperature (MAT), MAP, warming magnitude, warming duration, latitude and elevation, even when grouped by different warming magnitudes (Supplementary Figs 1–3).

### Temperature sensitivity

**Figure 5:**
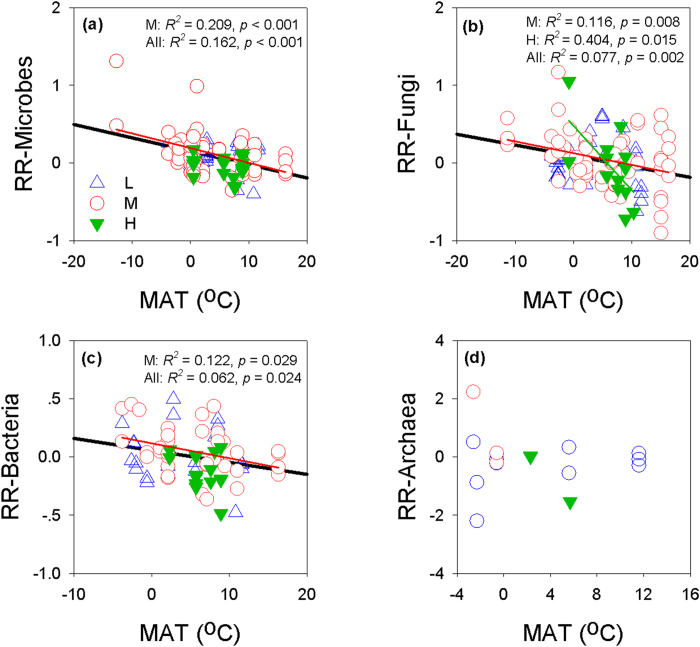
Relationships between the response ratios (RR) of the abundance of microbes (a), fungi (b), bacteria (c), and Archaea (d) and mean annual temperature (MAT). L: low warming magnitude; M: medium warming magnitude; H: high warming magnitude; the black solid line show the overall relationship between the MAT and RR of microbial abundances to warming.

We found significant negative relationships between the RRs of microbial, fungal and bacterial abundances and MAT (Fig. 5 and Supplementary Fig. 2). For microbial and fungal abundances, these negative relationships still held true when the results from the coldest site, or the warmest site, or both sites were excluded. For bacterial abundance, this relationship remained significant when the results from the warmest region were excluded, but not when the results from coldest region were excluded. When grouped by warming magnitudes, significant relationships were found between the RRs of microbial, fungal and bacterial abundances and MAT for medium warming magnitude, and for fungal abundance under high warming magnitude. In addition, we did not detect any relationships between warming magnitude and MAT (Supplementary Fig. 4)

### Soil respiration

**Figure 6:**
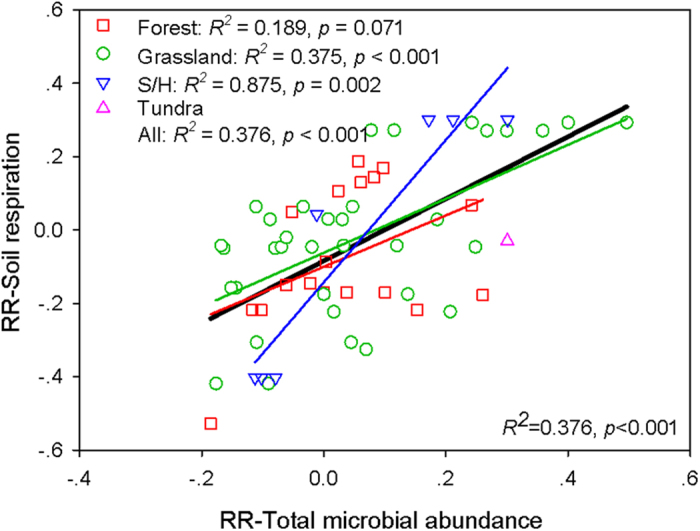
Relationships between the response ratios (RR) of total microbial abundance and RR of soil respiration (SR). S/H: shrubland/heathland, the black solid line show the overall relationship between the RR of soil respiration and RR of total microbial abundance.

A subset of studies included in this meta-analysis reported the effects of warming on SR in addition to the effects of warming on microbial, fungal and bacterial abundance. We found significant positive relationships between the RR of total microbial abundance and SR, this relationship still hold true when grouped by vegetation types and SR measurement methods. When these analyses were grouped by warming magnitudes, significant relationships were found for low and medium warming magnitudes, but not for the high warming magnitude (Fig. 6 and Supplementary Fig. 5).

## Discussion

### Warming on microbial abundances

Our meta-analysis showed that warming had positive effect on microbial abundance and this effect tended to be stronger in colder regions (Figs 1 and 5 and Supplementary Fig. 2). A recent incubation study that involved the samples from wide-range of MAT gradients also showed that the stronger warming effects on microbes occurred in colder regions^15^. Microbes can adapt to low temperatures at the cellular level by reducing metabolic activities or by becoming dormant^19^, but in either cases, there would be competitive advantages to rapidly respond to rising temperatures^23^,^24^. This thermal sensitivity of microbial abundance suggest that climate warming would have non-uniform effects on microbial associated ecosystem functions, and this information should be incorporated into ESMs.

We also detected significant positive relationships between the RRs of dissolved organic carbon (DOC), soil labile nitrogen (SLN), soil total nitrogen (STN) and RR of microbial abundance, as well as significant negative relationships between the RRs of microbial, fungal, and bacterial abundances and substrate C: N ratio (Supplementary Fig. 6). This substrate-regulated the effects of warming on microbial abundances has been reported elsewhere^15^,^20^,^25^. Indeed, colder regions historically accumulate large amounts of SOM due to the thermodynamic constraints imposed by low temperatures^26^,^27^. The richer substrate in these regions would facilitate the stronger warming effects on microbial abundance in these colder regions when the temperature increased. This was mainly due to the warming-induced SOM decomposition in these regions would provide more substrates to further fuel microbial metabolic activities^26^,^27^.

It should be noted that warming magnitude may affect the relationships between the RR of microbial abundances and MAT (Fig. 5, Supplementary Figs 1 to 3, 5 and 6). Thus, one may reasonably concern that if more lower warming magnitude treatments occurred in colder regions, and then our key conclusion would be weakened. We therefore did a further regression to test how warming magnitudes were distributed along MAT, fortunately, we did not find any clear relationship between warming magnitude and MAT (Supplementary Fig. 4). These results suggest that despite warming magnitude may affect the relationships between the RR of microbial abundance and MAT, our key conclusion of stronger warming effects on microbial abundances in colder regions still hold true.

Archaea often were extremophiles living in harsh environments, and thus the Archaea abundance tended to be resistant to climate warming^1^,^28^,^29^. In addition, a subset of selected papers also showed that warming had significant effects on Actinomycetes and saprotrophic fungal abundance, but had no effects on others (Table 1). Changes in specific microbial groups could have significant implications for total microbial abundance^3^,^30^,^31^. Our knowledge of microbial abundance would benefit greatly from better understanding of soil specific microbial groups.

### Microbial abundance and soil respiration

In support of our hypothesis, we found significant positive relationships between the RRs of SR and total microbial abundance (Fig. 6). These relationships were independent of vegetation types and SR measurement methods (Supplementary Fig. 5). Warming significantly stimulated microbial abundance as well as it synchronously accelerated decomposition^32^. This was further supported by our regression analysis that there were significant or marginally significant positive relationships between the RR of total microbial abundance and the RRs of phosphatase, glucosidase, phenol oxidase and N-Acetylglucosamine concentrations (Supplementary Fig. 6). Enhanced soil extracellular enzymes concentrations were expected to accelerate the degradation of litter and SOM, and that would in turn stimulate SR^33^,^34^. Consistently, three recent meta-analyses also demonstrated that warming significantly enhanced SR globally^32^,^35^,^36^. Our results therefore imply that the vast amounts of C accumulated in colder regions could be particular vulnerable to climate warming.

In addition, when grouped by warming magnitudes, this kind of significant positive relationship was not found for high warming magnitudes (Supplementary Fig. 5). This was partly due to the high warming magnitude induced water limitation may constrain the responses of microbial abundance and SR, or this was partly due to the publication bias (only 8 groups of data from the high warming magnitudes). Our results suggest that warming magnitude may affect the observed positive relationship between the RR of total microbial abundances and RR of SR.

### Vegetation and soil types

Warming had positive effects on microbial, bacterial and fungal abundances in tundras and histosols (Figs 1, 2, 3). The preliminary explanation should be attributed to the low MAT in tundras (-2.4 °C) and histosols (3.1 °C) in the current study, which were consistent with the discussion above. Secondly, litter produced from plants in the tundras were decomposed faster than litter of woody and evergreen plants^37^,^38^, and histosols stored at least one third of terrestrial SOM^39^,^40^. These properties could eventually help bringing relative higher soil substrate availability and thus facilitate the positive microbial responses^39^. Our results therefore indicate that these ecosystems and warming-induced ecosystem shifting in this way could be potential C emissions hotspots in the future warming scenarios^41^,^42^.

Warming also significantly enhanced microbial abundance in grasslands, while decreased bacterial abundance in forests (Figs 1 and 3). This could be related to the relatively higher allocation of plant productivity to belowground in grasslands will bring more SOM inputs to the soils compared with other biomes^43^,^44^,^45^. We also detected significant negative responses of fungal and bacterial abundance in podzols (Figs 2 and 3), which soils were characterized by low nutrient concentrations, low soil-water holding capacities, and low pH. It is thus likely that the effects of warming on microbial abundance in podzols are constrained by these factors. When re-analyzed these differential responses in different warming magnitudes, we found that these negative responses were mainly resulted form the relative higher warming magnitudes with significant negative effects on fungal and bacterial abundances (Supplementary Tables 1 to 3). For example, 90.5% and 87.5% of bacterial abundances in forests and podzols were from the relative higher warming magnitudes. These results suggest that warming magnitudes may affect these differential responses of microbial abundances to warming among vegetation and soil types.

### Warming protocols

We found significant negative relationships between warming magnitudes and RRs of microbial, fungal and bacterial abundances (Supplementary Fig. 1). Higher warming magnitudes were often with greater reductions in soil moisture, and thus had negative effects on soil microbial abundance (Supplementary Fig. 8). Soil warming by heating-cables were often with higher warming magnitudes and more pronounced reductions in soil moisture^35^. Whereas, OTC and curtains were usually associated with lower warming magnitudes and smaller negative effects on soil moisture. Our results indicate that warming magnitudes and warming methods should be considered when evaluating the effects of warming on microbial abundance.

No clear relationships were observed between the RR of microbial abundance and warming duration. One should interpret this with caution since only 11% of the observations involved warming treatments lasting more than 10 years. If warming were extended, neutral or negative responses might well be possible due to the depletion of substrate availability and limitation of soil moisture^7^,^41^. Therefore, our syntheses emphasize the necessity for long warming duration experiments. Diurnal and day warming had positive effects on microbial abundance, but not for night warming (Fig. 1). One possible consideration was that day warming promoted the C allocation to belowground, while night warming led to limitations of these substrates^46^,^47^.

In conclusion, warming had stronger impacts on microbial abundance in colder than warm regions, and there was a significant positive relationship between the RRs of SR and total microbial abundance. These results indicate that the large quantities of C stocked in colder regions could be more vulnerable than currently projected, and models should take the thermal sensitivity of microbial abundance into consideration when they are used for projecting future climate-carbon cycle feedbacks.

## Methods

### Source of data

We searched journal articles published before 2015 using the Web of Science (http://apps.webofknowledge.com/) and China Knowledge Resource Integrated Database (http://www.cnki.net/). Briefly, the following keywords and combinations were used for the searching: (1) “climate change” or “warming” or “temperature”; (2) “biota” or “microbe” or “microbial” or “fungi” or “bacterial” or “Archaea”; and (3) “terrestrial” or “soil” or “land”.

Based on the methods for meta-analysis^48^, studies were selected according to the following criteria: (1) all results were from field experiments. Specifically, we limited our data collections to studies ≥1 yr; (2) control and warming treatments had to be made at the same experimental sites. This allowed us to exclude variations induced by microclimate or vegetation or soil types; (3) data collection was limited to results in which means, stand deviations (SDs), and replicate numbers were reported. If standard errors (SEs) were reported, the following equation was used to calculate SD


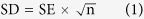


where n was the replicate numbers; (4) warming protocols (warming methods, warming magnitude, warming season, warming time, and warming duration) had to be clearly described or accessible from the cited articles; (5) if more than one field manipulation experiment were reported in the same article but with different environmental variables or vegetation types or soil types (e.g. experiments conducted under various geographical location or microclimate), each was regarded as an independent study; (6) if multiple measurements were measured in the same year, we only chose the last set of measurements; and (7) if the results were reported from different soil layers, we only included the results from the uppermost soil layer.

### Data acquisition

In total, 64 published papers were selected from 45 study sites (Supplementary Fig. 9). For each selected paper, we recorded microbial, fungal, bacterial, and Archaea abundance or biomass. Meanwhile, we also recorded study site, latitude, longitude, elevation, MAT, MAP, vegetation types, soil types (http://www.fao.org), warming methods (infrared-heaters, OTC, green house, heating-cables, and curtains), warming time (day, night, and diurnal), warming season (all-year and growing season) from the selected papers or cited papers. When possible, DOC, SLN, STN, substrate C: N, soil extracellular enzymes, soil moisture, and SR were also recorded. We defined SR as the amount of soil CO_2_ release measured by soil chambers in the field studies or during laboratory incubations. This method has been successfully used in two previous meta-analyses to evaluate the responses of microbial abundance and SR to other global climate change factors^21^,^22^. If data were presented graphically, we used Engauge Digitizer 4.1 (http://digitizer.sourceforge.net) to digitize the data. For some of environmental variables that could not be acquired from the selected papers and the cited papers, we extracted these data from a global data base (http://www.worldclim.org/) using location information (latitude and longitude). If critical information could not be directly acquired from the selected articles or cited articles, the authors were contacted.

### Microbial measurements

In the current meta-analysis, multiple types of microbial measurements were considered. Total microbial biomass or abundance were determined by CF^49^, or PLFA^50^. Fungal abundance was measured by microscopy, fungi PLFA, concentrations of ergosterol, or quantitative polymerase chain reaction analysis (qPCR). For bacteria, microscopy, bacteria PLFA, and qPCR were adopted, while for Archaea, the results were obtained by qPCR.

### Data analysis

Meta-analysis approach was used to determine the significance of microbial responses to a variety of experimental warming treatments^21^,^22^,^32^,^48^,^51^. For each study, the response ratio (RR) was calculated as below:


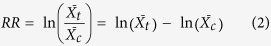


where 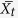 and 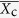 were means of warming and control treatments, respectively. The distribution of the RRs calculated in this way was typically nearly normal and the biases were minor^48^. The variance within each study was calculated using the means, replicate numbers, and SDs of both warming and control treatments. Details concerning the methods for calculating the variance can be found in^32^,^48^,^51^.

We used the MetaWin software (Sinauer AsSOMiates Inc., Sunderland, MA, USA) to calculate overall weighted response ration (RR_++_) and 95% bootstrap confidence intervals (CIs) for the all dataset and the grouped dataset. Significant responses (*p* < 0.05) were determined if CIs of RR_++_ did not overlap with 0. Warming-induced changes for a certain categorical group were calculated by


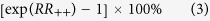


Random effects models in the meta-analysis were used to compare differences among groups in the ways similar to the analysis of variance framework. We sequentially compared RR_++_ among measurements, vegetation types, soil types, and warming protocols. A linear regression analysis was adopted to examine the relationships between the RRs of microbial, fungal, bacterial, and Archaea abundances and MAT, MAP, warming magnitude, warming duration, latitude, elevation, RRs of soil moisture, RRs of enzymatic concentrations, and RRs of SR. To test the impacts induced by warming magnitudes, we also did regression analysis for each similar warming magnitudes for all of the above regression analysis, respectively. The warming magnitude classification are consistent with previous meta-analysis^32^, in which the warming magnitude are grouped by low warming magnitude (<1 °C), medium warming magnitude (1~3 °C), and high warming magnitude (>3 °C).

Total heterogeneity (*Q*_T_) was divided into within groups (*Q*_W_) and between-group (*Q*_B_) heterogeneities. For each categorical group, significant between-group difference was determined at *p* < 0.05. Due to the preference for publishing larger effects than smaller ones, we used Kendall’s tau rank and Spearman’s rank correlation to infer publication bias^21^,^52^,^53^. All the methods in the present meta-analysis have been successfully used in numerous previous studies^21^,^51^,^53^.

## Additional Information

**How to cite this article**: Chen, J. *et al*. Stronger warming effects on microbial abundances in colder regions. *Sci. Rep*. **5**, 18032; doi: 10.1038/srep18032 (2015).

## Supplementary Material

Supplementary Information

## Figures and Tables

**Table 1:** Microbial abundance and publication bias to experimental warming.

Group	n	RR_++_	Bootstrap CI	Kendall’s tau rank	Spearman rank
Microbes*	110	0.073	0.0389~0.1045	0.70018	0.71076
Fungi	120	0.0298	−0.0265~0.085	0.93648	0.96853
Bacteria	82	0.0113	−0.03~0.0531	0.98518	0.99266
Archaea	17	−0.0754	−0.2991~0.1258	0.81467	0.80809
**Specific microbial groups**					
Gram positive bacteria	39	−0.0053	−0.063~0.0593	**0.02997**	**0.03449**
Gram negative bacteria	35	0.0232	−0.0549~0.1107	0.18435	0.27081
Actinomycetes*	14	0.1045	0.019~0.2012	0.58964	0.60127
Arbuscular mycorrhizal fungi	30	0.0276	-0.086~0.1307	0.90306	0.84389
Saprotrophic fungi *	5	0.1909	0.1124~0.26	n.a.	n.a.
Bacteria: Fungi*	85	0.069	0.0078~0.1221	0.75148	0.8572
GN: GP	41	−0.0291	−0.0676~0.0146	0.65982	0.612

^*^Significant warming effect on group (*p* < 0.05). Boldface for the Kendall’s tau rank and Spearman rank indicate significant publication bias at *p* < 0.05. n: the number of studies included for the meta-analysis; RR_++_: weighted response ratio; CI: bootstrap confidence interval; GN: gram negative bacteria; GP: gram positive bacteria. See Fig. 1 for other information.
