# Supplementary material for: Stronger warming effects on microbial abundances in colder regions
Source: Sci Rep. 2015 Dec 10;5:18032. doi: 10.1038/srep18032 (PMC4674839; doi:10.1038/srep18032)
Supplement: Supplementary Information [file srep18032-s1.doc]

**Stronger warming effects on microbial abundances in colder regions**

Ji Chen^1,2^, Yiqi Luo^3,4*^, Jianyang Xia^3^, Lifen Jiang^3^, Xuhui Zhou^5^, Meng Lu^5^, Junyi Liang^3^, Zheng Shi^3^, Shelby Shelton^3^, and Junji Cao^1,6*^

^1^State Key Laboratory of Loess and Quaternary Geology (SKLLQG), and Key Laboratory of Aerosol Chemistry and Physics, Institute of Earth Environment, Chinese Academy of Sciences, Xi’an 710061, China, ^2^University of Chinese Academy of Sciences, Beijing, 100049, China, ^3^University of Oklahoma, Department of Microbiology and Plant Biology, Norman, 73019, USA, ^4^Tsinghua University, Center for Earth System Science, Beijing, 100084, China, ^5^Fudan University, Coastal Ecosystems Research Station of Yangtze River Estuary, Ministry of Education Key Laboratory for Biodiversity Science and Ecological Engineering, The Institute of Biodiversity Science, Shanghai 200433, China, ^6^ Xi’an Jiaotong University, Institute of Global Environmental Change, Xi’an 710049, China.

*Corresponding author:

Junji Cao, cao@loess.llqg.ac.cn, Tel: +86-29-62336233, Fax: +86-29-62336234;

Yiqi Luo, yluo@ou.edu, Tel: +1-405-325-1651, Fax: +1-405-325-7619.

**Supplementary Notes**

(i) **Supplementary tables and figures**

**Supplementary Table 1.** The effects of warming on microbial abundance in different warming magnitudes.

| Group | n | RR_++_ | CI | Percentage (%) | Low warming magnitude | | |  | Medium warming magnitude | | |  | High warming magnitude | | |
| --- | --- | --- | --- | --- | --- | --- | --- | --- | --- | --- | --- | --- | --- | --- | --- |
|  |  |  |  |  | n | RR_++_ | CI |  | n | RR_++_ | CI |  | n | RR_++_ | CI |
| All | **110** | **0.073** | **0.039 ~ 0.105** | **7.573** | **31** | **0.068** | **0.027 ~ 0.106** |  | **63** | **0.109** | **0.062 ~ 0.156** |  | 16 | -0.046 | -0.125 ~ 0.025 |
|  |  |  |  |  |  |  |  |  |  |  |  |  |  |  |  |
| PLFA | **36** | **0.082** | **0.017 ~ 0.147** | **8.546** | 8 | 0.091 | -0.006 ~ 0.153 |  | **22** | **0.140** | **0.063 ~ 0.223** |  | **6** | **-0.137** | **-0.241 ~ -0.055** |
| CF | **74** | **0.068** | **0.034 ~ 0.107** | **7.037** | **23** | **0.059** | **0.018 ~ 0.104** |  | **41** | **0.079** | **0.020 ~ 0.141** |  | 10 | 0.033 | -0.056 ~ 0.087 |
|  |  |  |  |  |  |  |  |  |  |  |  |  |  |  |  |
| Tundra | **24** | **0.140** | **0.067 ~ 0.219** | **15.027** | 5 | 0.085 | -0.203 ~ 0.227 |  | **15** | **0.180** | **0.093 ~ 0.297** |  | 4 | 0.004 | -0.120 ~ 0.119 |
| S/H | 4 | 0.042 | -0.063 ~ 0.127 | 4.289 | 2 | -- | -- |  | 2 | -- | -- |  | -- | -- | -- |
| Grassland | **44** | **0.080** | **0.023 ~ 0.143** | **8.329** | **18** | **0.084** | **0.020 ~ 0.141** |  | 26 | 0.073 | -0.014 ~ 0.177 |  | -- | -- | -- |
| Forest | 38 | 0.034 | -0.01 ~ 0.077 | 3.458 | 6 | 0.044 | -0.001 ~ 0.076 |  | **20** | **0.096** | **0.028 ~ 0.162** |  | 12 | -0.053 | -0.152 ~ 0.027 |
|  |  |  |  |  |  |  |  |  |  |  |  |  |  |  |  |
| Arenosols | 14 | 0.021 | -0.08 ~ 0.091 | 2.122 | 9 | 0.009 | -0.115 ~ 0.141 |  | 5 | 0.041 | -0.099 ~ 0.104 |  | -- | -- | -- |
| Cambisols | 32 | 0.047 | -0.004 ~ 0.100 | 4.812 | **23** | **0.069** | **0.006 ~ 0.12** |  | 6 | 0.030 | -0.056 ~ 0.196 |  | 3 | -0.043 | -0.130 ~ 0.064 |
| Histosols | **34** | **0.153** | **0.104 ~ 0.215** | **16.532** | **8** | **0.105** | **0.070 ~ 0.146** |  | **22** | **0.200** | **0.119 ~ 0.291** |  | 4 | 0.004 | -0.119 ~ 0.119 |
| Luvisol | 8 | 0.018 | -0.073 ~ 0.115 | 1.816 | 4 | 0.022 | -0.038 ~ 0.068 |  | 4 | 0.018 | -0.134 ~ 0.226 |  | -- | -- | -- |
| Podzols | 18 | -0.016 | -0.095 ~ 0.060 | -1.587 | 2 | 0.029 | -0.012 ~ 0.212 |  | 7 | 0.063 | -0.071 ~ 0.225 |  | 9 | -0.071 | -0.187 ~ 0.023 |
| Vertisols | 4 | 0.051 | -0.065 ~ 0.203 | 5.232 | 2 | -- | -- |  | 2 | -- | -- |  | -- | -- |  |
|  |  |  |  |  |  |  |  |  |  |  |  |  |  |  |  |
| IH | **49** | **0.045** | **0.005 ~ 0.085** | **4.603** | **12** | **0.079** | **0.019 ~ 0.128** |  | 31 | 0.042 | -0.021 ~ 0.099 |  | 6 | 0.004 | -0.073 ~ 0.076 |
| OTC | **31** | **0.141** | **0.090 ~ 0.206** | **15.142** | **11** | **0.003** | **0.002 ~ 0.006** |  | **20** | **0.002** | **0.001 ~ 0.012** |  | -- | -- | -- |
| GH | 16 | 0.089 | -0.002 ~ 0.194 | 9.308 | -- | -- | -- |  | 12 | 0.124 | -0.003 ~ 0.265 |  | 4 | 0.003 | -0.130 ~ 0.122 |
| HA | 6 | **-0.183** | **-0.283 ~ -0.025** | **-16.723** | -- | -- | -- |  | -- | -- | -- |  | **6** | **-0.191** | **-0.288 ~ -0.021** |
| Curtains | 8 | 0.045 | -0.050 ~ 0.179 | 4.603 | 8 | 0.045 | -0.050 ~ 0.179 |  | -- | -- | -- |  | -- | -- | -- |
|  |  |  |  |  |  |  |  |  |  |  |  |  |  |  |  |
| Night | 18 | -0.028 | -0.086 ~ 0.036 | -2.761 | 10 | 0.018 | -0.095 ~ 0.147 |  | 4 | -0.035 | -0.140 ~ 0.073 |  | **4** | **-0.064** | **-0.090 ~ -0.021** |
| Diurnal | **86** | **0.084** | **0.047 ~ 0.125** | **8.763** | **21** | **0.077** | **0.037 ~ 0.117** |  | **55** | **0.116** | **0.064 ~ 0.174** |  | 10 | -0.099 | -0.204 ~ 0.011 |
| Day | **6** | **0.167** | **0.114 ~ 0.248** | **18.175** | -- | -- | -- |  | **4** | **0.216** | **0.172 ~ 0.319** |  | 2 | -- | -- |
|  |  |  |  |  |  |  |  |  |  |  |  |  |  |  |  |
| All-year | **97** | **0.072** | **0.040 ~ 0.111** | **7.466** | **31** | **0.068** | **0.031 ~ 0.108** |  | **53** | **0.110** | **0.048 ~ 0.172** |  | 13 | -0.048 | -0.144 ~ 0.031 |
| GS | 13 | 0.080 | -0.023 ~ 0.160 | 8.329 | -- | -- | -- |  | **10** | **0.109** | **0.024 ~ 0.190** |  | 3 | -0.041 | -0.130 ~ 0.064 |

Note: PLFA: phospholipid fatty acids; CF: chloroform fumigation; S/H: shrubland/heathland; IH: infrared heater; OTC: open top chamber; GH: green house; HA: heating cable; GS: growing season; n: the number of studies included for the meta-analysis; RR_++_: weighted response ratio; CI: bootstrap confidence interval; Percentage: percentage is calculated by [exp(RR_++_)-1]×100%; boldface indicate significant warming effects on microbial abundance. For studies less than two, the results are not shown. Warming magnitudes are classified based on previous meta-analysis (Lu *et al.* 2013), low warming magnitude (< 1 ^o^C), medium warming magnitude (1~3 ^o^C), high warming magnitude (> 3 ^o^C).

**Supplementary Table 2.** The effects of warming on fungal abundance in different warming magnitudes.

| Group | n | RR_++_ | CI | Percentage (%) | Low warming magnitude | | |  | Medium warming magnitude | | |  | High warming magnitude | | |
| --- | --- | --- | --- | --- | --- | --- | --- | --- | --- | --- | --- | --- | --- | --- | --- |
|  |  |  |  |  | n | RR_++_ | CI |  | n | RR_++_ | CI |  | n | RR_++_ | CI |
| All | 120 | 0.030 | -0.027 ~ 0.085 | 3.045 | 46 | 0.036 | -0.036 ~ 0.112 |  | 60 | 0.055 | -0.021 ~ 0.137 |  | 14 | -0.100 | -0.332 ~ 0.147 |
|  |  |  |  |  |  |  |  |  |  |  |  |  |  |  |  |
| PLFA | 62 | 0.002 | -0.070 ~ 0.074 | 0.200 | 18 | 0.079 | -0.081 ~ 0.233 |  | 34 | 0.006 | -0.071 ~ 0.086 |  | 10 | -0.148 | -0.353 ~ 0.071 |
| Ergosterol | 32 | 0.050 | -0.063 ~ 0.162 | 5.127 | 9 | -0.028 | -0.092 ~ 0.041 |  | 20 | 0.106 | -0.015 ~ 0.230 |  | 3 | 0.019 | -0.631 ~ 1.049 |
| qPCR | 23 | 0.101 | -0.024 ~ 0.258 | 10.628 | 19 | 0.030 | -0.079 ~ 0.131 |  | **3** | **0.699** | **0.073 ~ 1.168** |  | 1 | -- | -- |
| Microscope | 3 | -0.178 | -0.421 ~ 0.019 | -16.306 | -- | -- | -- |  | 3 | -0.178 | -0.421 ~ 0.019 |  | -- | -- | -- |
|  |  |  |  |  |  |  |  |  |  |  |  |  |  |  |  |
| Tundra | **23** | **0.091** | **0.021 ~ 0.173** | **9.527** | 9 | -0.026 | -0.093 ~ 0.042 |  | **14** | **0.149** | **0.047 ~ 0.246** |  | -- | -- | -- |
| S/H | 10 | 0.066 | -0.041 ~ 0.183 | 6.823 | 4 | 0.040 | -0.144 ~ 0.179 |  | 6 | 0.072 | -0.013 ~ 0.211 |  | -- | -- | -- |
| Grassland | 60 | 0.022 | -0.056 ~ 0.103 | 2.224 | 32 | 0.052 | -0.058 ~ 0.154 |  | 27 | 0.010 | -0.096 ~ 0.132 |  | 1 | -- | -- |
| Forest | 27 | -0.031 | -0.198 ~ 0.143 | -3.052 | 1 | -- | -- |  | 13 | 0.231 | -0.280 ~ 0.231 |  | 13 | 0.218 | -0.241 ~ 0.218 |
|  |  |  |  |  |  |  |  |  |  |  |  |  |  |  |  |
| Arenosols | 12 | 0.124 | -0.002 ~ 0.238 | 13.202 | 5 | 0.134 | -0.184 ~ 0.343 |  | **7** | **0.135** | **0.020 ~ 0.247** |  | -- | -- | -- |
| Cambisols | 18 | 0.007 | -0.142 ~ 0.160 | 0.702 | 8 | 0.092 | -0.072 ~ 0.242 |  | 5 | 0.035 | -0.226 ~ 0.396 |  | 5 | -0.233 | -0.486 ~ 0.050 |
| Histosols | **47** | **0.124** | **0.034 ~ 0.221** | **13.202** | **20** | **0.113** | **0.015 ~ 0.217** |  | 25 | 0.106 | -0.023 ~ 0.261 |  | 2 | -- | -- |
| Kastanozems | 9 | -0.061 | -0.387 ~ 0.222 | -5.918 | -- | -- | -- |  | 9 | -0.061 | -0.387 ~ 0.222 |  | -- | -- | -- |
| Luvisol | 16 | -0.031 | -0.102 ~ 0.041 | -3.052 | **6** | **-0.119** | **-0.190 ~ -0.050** |  | 10 | -0.007 | -0.089 ~ 0.119 |  | -- | -- | -- |
| Podzols | **8** | **-0.235** | **-0.421 ~ -0.050** | **-20.943** | 1 | -- | -- |  | 1 | -- | -- |  | **6** | **-0.237** | **-0.422 ~ -0.052** |
| Vertisols | 8 | -0.144 | -0.394 ~ 0.113 | -13.411 | **4** | **-0.307** | **-0.447 ~ -0.088** |  | 3 | -0.184 | -0.441 ~ 0.249 |  | 1 | -- | -- |
|  |  |  |  |  |  |  |  |  |  |  |  |  |  |  |  |
| Infrad-heaters | 55 | -0.030 | -0.111 ~ 0.062 | -2.955 | 22 | -0.013 | -0.152 ~ 0.129 |  | 26 | -0.017 | -0.102 ~ 0.06 |  | 7 | -0.149 | -0.479 ~ 0.278 |
| OTC | **28** | **0.129** | **0.031 ~ 0.256** | **13.769** | **12** | **0.115** | **0.040 ~ 0.190** |  | 16 | 0.134 | -0.037 ~ 0.361 |  | -- | -- | -- |
| Green-house | 25 | 0.070 | -0.027 ~ 0.176 | 7.251 | 8 | -0.030 | -0.104 ~ 0.041 |  | **17** | **0.200** | **0.069 ~ 0.303** |  | -- | -- | -- |
| Heating-cables | 7 | -0.025 | -0.227 ~ 0.234 | -2.469 | -- | -- | -- |  | -- | -- | -- |  | 7 | -0.025 | -0.227 ~ 0.234 |
| Curtains | **4** | **0.267** | **0.173 ~ 0.385** | **30.604** | **4** | **0.267** | **0.173 ~ 0.385** |  | -- | -- | -- |  | -- | -- | -- |
|  |  |  |  |  |  |  |  |  |  |  |  |  |  |  |  |
| Night | 14 | -0.092 | -0.255 ~ 0.062 | -8.789 | 8 | 0.031 | -0.162 ~ 0.253 |  | 2 | -- | -- |  | 4 | -0.268 | -0.571 ~ 0.016 |
| Diurnal | 105 | 0.045 | -0.014 ~ 0.106 | 4.603 | 37 | 0.037 | -0.041 ~ 0.119 |  | 58 | 0.058 | -0.021 ~ 0.138 |  | 10 | -0.002 | -0.285 ~ 0.338 |
|  |  |  |  |  |  |  |  |  |  |  |  |  |  |  |  |
| All-year | 81 | 0.018 | -0.053 ~ 0.096 | 1.816 | 30 | 0.071 | -0.037 ~ 0.186 |  | 43 | 0.028 | -0.071 ~ 0.135 |  | 8 | -0.214 | -0.473 ~ 0.031 |
| GS | 37 | 0.049 | -0.024 ~ 0.128 | 5.022 | 16 | -0.031 | -0.090 ~ 0.025 |  | 15 | 0.101 | -0.024 ~ 0.213 |  | 6 | 0.183 | -0.118 ~ 0.653 |

**Supplementary Table 3.** The effects of warming on bacterial abundance in different warming magnitudes.

| Group | n | RR_++_ | CI | Percentage (%) | Low warming magnitude | | |  | Medium warming magnitude | | |  | High warming magnitude | | |
| --- | --- | --- | --- | --- | --- | --- | --- | --- | --- | --- | --- | --- | --- | --- | --- |
|  |  |  |  |  | n | RR_++_ | CI |  | n | RR_++_ | CI |  | n | RR_++_ | CI |
| All | 82 | 0.011 | -0.030 ~ 0.053 | 1.106 | 28 | 0.009 | -0.057 ~ 0.079 |  | 39 | 0.053 | -0.005 ~ 0.118 |  | **15** | **-0.100** | **-0.187 ~ -0.031** |
|  |  |  |  |  |  |  |  |  |  |  |  |  |  |  |  |
| PLFA | 52 | -0.013 | -0.064 ~ 0.036 | -1.292 | 9 | -0.027 | -0.130 ~ 0.085 |  | 31 | 0.034 | -0.029 ~ 0.101 |  | **12** | **-0.163** | **-0.241 ~ -0.085** |
| qPCR | 25 | 0.058 | -0.003 ~ 0.127 | 5.971 | 17 | 0.042 | -0.036 ~ 0.134 |  | **5** | **0.192** | **0.024 ~ 0.346** |  | 3 | 0.024 | -0.004 ~ 0.060 |
| Microscope | 4 | 0.097 | -0.270 ~ 0.341 | 10.186 | 1 | -- | -- |  | 3 | 0.070 | -0.227 ~ 0.319 |  | -- | -- | -- |
|  |  |  |  |  |  |  |  |  |  |  |  |  |  |  |  |
| Tundra | **4** | **0.315** | **0.191 ~ 0.409** | **37.026** | 1 | -- | -- |  | **3** | **0.313** | **0.194 ~ 0.410** |  | -- | -- | -- |
| S/H | 10 | 0.078 | -0.077 ~ 0.198 | 8.112 | 4 | -0.154 | -0.211 ~ 0.011 |  | **6** | **0.151** | **0.021 ~ 0.273** |  | -- | -- | -- |
| Grassland | 47 | 0.014 | -0.028 ~ 0.056 | 1.410 | 21 | 0.010 | -0.049 ~ 0.083 |  | 26 | 0.017 | -0.036 ~ 0.074 |  | -- | -- | -- |
| Forest | **21** | **-0.098** | **-0.175 ~ -0.036** | **-9.335** | 2 | -- | -- |  | **4** | **-0.146** | **-0.304 ~ -0.003** |  | **15** | **-0.089** | **-0.179 ~ -0.024** |
|  |  |  |  |  |  |  |  |  |  |  |  |  |  |  |  |
| Arenosols | 12 | 0.028 | -0.051 ~ 0.113 | 2.840 | 5 | 0.044 | -0.262 ~ 0.262 |  | 7 | 0.024 | -0.052 ~ 0.106 |  | -- | -- | -- |
| Cambisols | 13 | -0.045 | -0.172 ~ 0.091 | -4.400 | 2 | -- | -- |  | **5** | **-0.151** | **-0.297 ~ -0.072** |  | 6 | -0.039 | -0.227 ~ 0.006 |
| Histosols | **20** | **0.125** | **0.048 ~ 0.208** | **13.315** | 9 | -0.001 | -0.100 ~ 0.095 |  | **11** | **0.209** | **0.104 ~ 0.312** |  | -- | -- | -- |
| Luvisol | 18 | -0.024 | -0.079 ~ 0.032 | -2.371 | 6 | -0.050 | -0.123 ~ 0.032 |  | 12 | -0.009 | -0.085 ~ 0.063 |  | -- | -- | -- |
| Podzols | **8** | **-0.154** | **-0.259 ~ -0.048** | **-14.273** | 1 | -- | -- |  | 1 | -- | -- |  | **6** | **-0.157** | **-0.253 ~ -0.047** |
| Vertisols | 9 | -0.030 | -0.142 ~ 0.111 | -2.955 | **5** | **-0.073** | **-0.097 ~ -0.06** |  | 3 | 0.016 | -0.321 ~ 0.435 |  | 1 | -- | -- |
|  |  |  |  |  |  |  |  |  |  |  |  |  |  |  |  |
| Infrad-heaters | 40 | -0.035 | -0.080 ~ 0.011 | -3.439 | 11 | -0.065 | -0.126 ~ 0.001 |  | 25 | 0.001 | -0.051 ~ 0.053 |  | **4** | **-0.185** | **-0.355 ~ -0.036** |
| OTC | **23** | **0.144** | **0.059 ~ 0.225** | **15.488** | 13 | 0.056 | -0.065 ~ 0.174 |  | **10** | **0.225** | **0.123 ~ 0.332** |  | -- | -- | -- |
| Green-house | 3 | -0.077 | -0.273 ~ 0.037 | -7.411 | -- | -- | -- |  | 3 | -0.077 | -0.273 ~ 0.037 |  | -- | -- | -- |
| Heating-cables | **11** | **-0.064** | **-0.147 ~ -0.003** | **-6.200** | -- | -- | -- |  | -- | -- | -- |  | **11** | **-0.064** | **-0.147 ~ -0.003** |
| Curtains | 4 | **0.197** | **0.073 ~ 0.291** | **21.774** | **4** | **0.197** | **0.073 ~ 0.291** |  | -- | -- | -- |  | -- | -- | -- |
|  |  |  |  |  |  |  |  |  |  |  |  |  |  |  |  |
| Night | 16 | -0.046 | -0.154 ~ 0.057 | -4.496 | 8 | -0.053 | -0.180 ~ 0.135 |  | 4 | 0.098 | -0.018 ~ 0.154 |  | **4** | **-0.186** | **-0.321 ~ -0.023** |
| Diurnal | 65 | 0.023 | -0.018 ~ 0.068 | 2.327 | 19 | 0.025 | -0.046 ~ 0.100 |  | 35 | 0.048 | -0.018 ~ 0.116 |  | **11** | **-0.067** | **-0.148 ~ -0.007** |
|  |  |  |  |  |  |  |  |  |  |  |  |  |  |  |  |
| All-year | 66 | 0.019 | -0.024 ~ 0.066 | 1.918 | 26 | 0.019 | -0.041 ~ 0.091 |  | 33 | 0.050 | -0.004 ~ 0.106 |  | **7** | **-0.159** | **-0.266 ~ -0.056** |
| GS | 14 | -0.029 | -0.154 ~ 0.085 | -2.858 | 2 | -- | -- |  | 4 | 0.070 | -0.262 ~ 0.316 |  | 8 | -0.056 | -0.159 ~ 0.006 |

**Supplementary Table 4.** The effects of warming on Archaea abundance in different warming magnitudes.

| Group | n | RR_++_ | CI | Percentage (%) | Low warming magnitude | | |  | Medium warming magnitude | | |  | High warming magnitude | | |
| --- | --- | --- | --- | --- | --- | --- | --- | --- | --- | --- | --- | --- | --- | --- | --- |
|  |  |  |  |  | n | RR_++_ | CI |  | n | RR_++_ | CI |  | n | RR_++_ | CI |
| All | 17 | -0.075 | -0.299 ~ 0.126 | -7.226 | 11 | -0.194 | -0.486 ~ 0.058 |  | 3 | 0.255 | -0.406 ~ 2.234 |  | 3 | -0.032 | -1.540 ~ 0.031 |
|  |  |  |  |  |  |  |  |  |  |  |  |  |  |  |  |
| S/H | 5 | 0.000 | -0.330 ~ 0.325 | 0.000 | 3 | -0.031 | -0.406 ~ 0.406 |  | 2 | 0.047 | -0.406 ~ 0.406 |  | -- | -- | -- |
| Grassland | 9 | -0.183 | -0.598 ~ 0.319 | -16.723 | 8 | -0.138 | -0.655 ~ 0.393 |  | 1 | -- | -- |  | -- | -- | -- |
| Forest | 3 | -0.043 | -1.540 ~ 0.031 | -4.209 | -- | -- | -- |  | -- | -- | -- |  | 3 | -0.043 | -1.540 ~ 0.031 |
|  |  |  |  |  |  |  |  |  |  |  |  |  |  |  |  |
| Histosols | 11 | -0.084 | -0.467 ~ 0.264 | -8.057 | 8 | -0.251 | -0.720 ~ 0.111 |  | 3 | 0.357 | -0.406 ~ 2.234 |  | -- | -- | -- |
| Vertisols | 3 | -0.098 | -0.290 ~ 0.127 | -9.335 | 3 | -0.098 | -0.290 ~ 0.127 |  | -- | -- | -- |  | -- | -- | -- |
|  |  |  |  |  |  |  |  |  |  |  |  |  |  |  |  |
| Infrad-heaters | 3 | -0.098 | -0.290 ~ 0.127 | -9.335 | 3 | -0.098 | -0.290 ~ 0.127 |  | -- | -- | -- |  | -- | -- | -- |
| OTC | 11 | -0.084 | -0.468 ~ 0.270 | -8.057 | 8 | -0.251 | -0.697 ~ 0.136 |  | 3 | 0.357 | -0.406 ~ 2.234 |  | -- | -- | -- |
| Heating-cables | 3 | -0.046 | -1.540 ~ 0.031 | -4.496 | -- | -- | -- |  | -- | -- | -- |  | 3 | -0.046 | -1.540 ~ 0.031 |
|  |  |  |  |  |  |  |  |  |  |  |  |  |  |  |  |
| All-year | 10 | -0.154 | -0.523 ~ 0.218 | -14.273 | 9 | 0.128 | -0.529 ~ 0.271 |  | 1 | -- | -- |  | -- | -- | -- |
| GS | 5 | -0.051 | -0.326 ~ 0.204 | -4.972 | 2 | -0.121 | -0.406 ~ 0.406 |  | -- | -- | -- |  | 3 | -0.005 | -0.819 ~ 0.031 |

**Supplementary Figure 1.** Relationships between the response ratios (RR) of the abundances of microbes (a), fungi (b), bacteria (c), and Archaea (d) and warming magnitude. And relationships between RR of the abundance sof microbes (e), fungi (f), bacteria (g), and Archaea (h) and warming duration. Different colors in (e) to (h) show the results from different warming magnitudes.

**Supplementary Figure 2.** Relationships between the response ratios (RR) of the abundances of microbes (a), fungi (b), bacteria (c), and Archaea (d) and mean annual precipitation (MAP). And relationships between the binned RR of the abundances of microbes (e), fungi (f), bacteria (g), and Archaea (h) and mean annual temperature (MAT). Different colors in (a) to (d) show the results from different warming magnitudes. “Binned response ratio” is the mean response ratio of microbial abundance in each centigrade MAT.

**Supplementary Figure 3.** Relationships between the response ratios (RR) of the abundance of microbes (a), fungi (b), bacteria (c), and Archaea (d) and latitude. And relationships between the RR of the abundance of microbes (e), fungi (f), bacteria (g), and Archaea (h) and elevation. Different colors in (a) to (h) show the results from different warming magnitudes. Apart from the significant positive relationship between the RR fungal abundance and latitude for the medium warming magnitudes, no any other significant relationship is found between the RRs of microbial abundance and elevation and latitude, suggesting that elevation or latitude may not the controlling factors affecting the effects of warming on microbial abundances.

**Supplementary Figure 4.** Distribution of warming magnitude with mean annual air temperature. No any relationship is found between mean annual temperature and warming magnitude, suggesting that warming magnitude may randomly distributed across different MAT regions. This figure is used to support the stronger warming effects in colder regions might be independent of warming magnitude.

**Supplementary Figure 5.** Relationships between the response ratios (RR) of total microbial abundance and RR of soil respiration (SR) in different warming magnitude (a) and in different SR measurement methods (b). Significant positive relationships between the RR of total microbial abundance and RR of SR are found for low and medium warming magnitude. There also are significant relationships between the RR of total microbial abundance and RR of SR for both field and incubation studies. These results suggesting that warming magnitude and SR measurement methods may not the controlling factors affecting the observed significant positive relationship between the RR of total microbial abundance and RR of SR in figure 6.

**Supplementary Figure 6.** Relationships between the binned response ratios (RR) of the abundances of microbes (a), fungi (b), and bacteria (c) and substrate C: N. And relationships between the RR of the abundance of microbes and RR of soil dissolved organic carbon (DOC, d), soil labile nitrogen (SLN, b), and soil total nitrogen (STN, c). Different colors in (a) to (f) show the results from different warming magnitudes. There are significant relationships between the RR of microbial abundances and substrate C : N ratio, and there are significant relationship between the RRs of DOC, SLN, STN and RRs of microbial abundances, suggesting that substrate nutrient availability may affect the effects of warming on microbial abundances.

**Supplementary Figure 7.** Relationships between the response ratios (RR) of total microbial abundances and RRs of phosphatase (a), β-1,4-Glucosidase (b), phenol oxidase (c), and N-Acetylglucosamine (d). Different colors in (a) to (d) show the results from different warming magnitudes. There are significant or marginally significant positive relationships between the RR of total microbial abundance and the RRs of phosphatase, glucosidase, phenol oxidase and N-Acetylglucosamine concentrations. This figure is used to provide one possible mechanism associated with the significant positive relationship between the RR of SR and RR of microbial abundance.

**Supplementary Figure 8.** Relationships between the warming magnitude and response ratios (RR) of soil moisture (a). And the relationships between the RR of soil moisture and RR of microbial abundance (b). Different colors in (b) show the results from different warming magnitudes. Higher warming magnitude are significantly associated with more evident decrease in soil moisture (a), and the effects of warming on soil moisture are closely related to the effects of warming on microbial abundance. This figure is used to support the conclusion that a significant negative relationship was found between the RR of microbial abundance and warming magnitude.


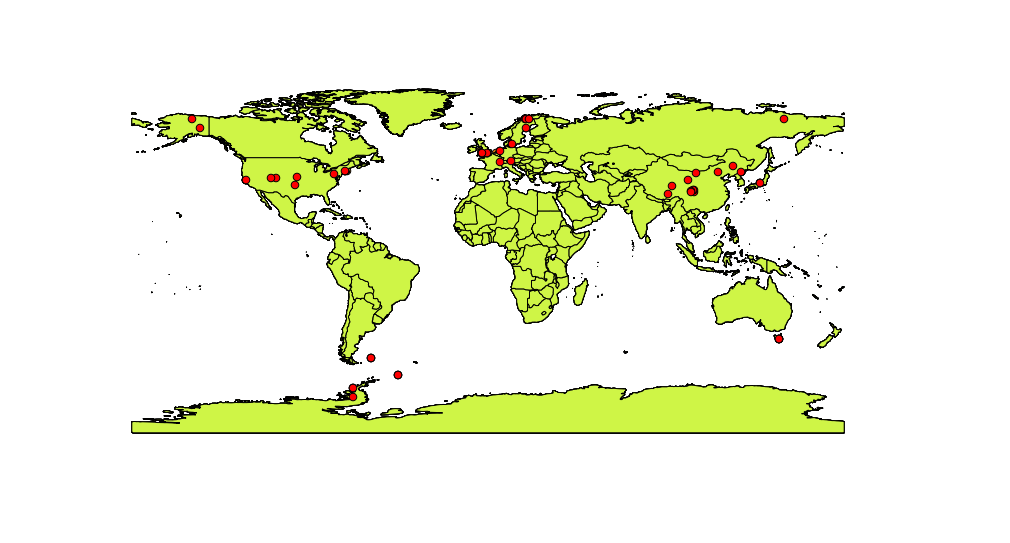


**Supplementary Figure 9.** Global distribution of warming experiments selected in this meta-analysis. Map was created with QGIS version 2.0.1 (Open Source Geospatial Foundation Project, [http://qgis.osgeo.org](http://qgis.osgeo.org/)). Global map was downloaded from natural earth (http://www.naturalearthdata.com/).

**(ii) Studies included in the current meta-analysis:**

1 Zhao, C. *et al.* Effects of experimental warming and nitrogen fertilization on soil microbial communities and processes of two subalpine coniferous species in Eastern Tibetan Plateau, China. *Plant Soil* **382**, 189-201, doi:10.1007/s11104-014-2153-2 (2014).

2 Zhang, B. *et al.* Responses of Soil Microbial Communities to Experimental Warming in Alpine Grasslands on the Qinghai-Tibet Plateau. *Plos One* **9**, doi:DOI 10.1371/journal.pone.0103859 (2014).

3 Jing, X. *et al.* No temperature acclimation of soil extracellular enzymes to experimental warming in an alpine grassland ecosystem on the Tibetan Plateau. *Biogeochemistry* **117**, 39-54, doi:DOI 10.1007/s10533-013-9844-2 (2014).

4 Wang, X. X. *et al.* Effects of short-term and long-term warming on soil nutrients, microbial biomass and enzyme activities in an alpine meadow on the Qinghai-Tibet Plateau of China. *Soil Biol Biochem* **76**, 140-142, doi:DOI 10.1016/j.soilbio.2014.05.014 (2014).

5 Rui, Y. C. *et al.* Warming and grazing affect soil labile carbon and nitrogen pools differently in an alpine meadow of the Qinghai-Tibet Plateau in China. *J Soil Sediment* **11**, 903-914, doi:DOI 10.1007/s11368-011-0388-6 (2011).

6 Shen, R. C., Xu, M., Chi, Y. G., Yu, S. & Wan, S. Q. Soil Microbial Responses to Experimental Warming and Nitrogen Addition in a Temperate Steppe of Northern China. *Pedosphere* **24**, 427-436 (2014).

7 Suseela, V., Tharayil, N., Xing, B. S. & Dukes, J. S. Warming alters potential enzyme activity but precipitation regulates chemical transformations in grass litter exposed to simulated climatic changes. *Soil Biol Biochem* **75**, 102-112, doi:DOI 10.1016/j.soilbio.2014.03.022 (2014).

8 Fu, G., Shen, Z. X., Zhang, X. Z. & Zhou, Y. T. Response of soil microbial biomass to short-term experimental warming in alpine meadow on the Tibetan Plateau. *Appl Soil Ecol* **61**, 158-160, doi:DOI 10.1016/j.apsoil.2012.05.002 (2012).

9 Rudgers, J. A. *et al.* Responses of high-altitude graminoids and soil fungi to 20 years of experimental warming. *Ecology* **95**, 1918-1928 (2014).

10 Zhou, X. Q. *et al.* Warming and increased precipitation have differential effects on soil extracellular enzyme activities in a temperate grassland. *Sci Total Environ* **444**, 552-558, doi:DOI 10.1016/j.scitotenv.2012.12.023 (2013).

11 Zhang, N. L. *et al.* Soil microbial responses to warming and increased precipitation and their implications for ecosystem C cycling. *Oecologia* **173**, 1125-1142, doi:DOI 10.1007/s00442-013-2685-9 (2013).

12 Yang, W. *et al.* The Arbuscular Mycorrhizal Fungal Community Response to Warming and Grazing Differs between Soil and Roots on the Qinghai-Tibetan Plateau. *Plos One* **8**, doi:ARTN e76447

DOI 10.1371/journal.pone.0076447 (2013).

13 Xu, G., Jiang, H., Zhang, Y. B., Korpelainen, H. & Li, C. Y. Effect of warming on extracted soil carbon pools of Abies faxoniana forest at two elevations. *Forest Ecol Manag* **310**, 357-365, doi:DOI 10.1016/j.foreco.2013.08.038 (2013).

14 Sistla, S. A. & Schimel, J. P. Seasonal patterns of microbial extracellular enzyme activities in an arctic tundra soil: Identifying direct and indirect effects of long-term summer warming. *Soil Biol Biochem* **66**, 119-129, doi:DOI 10.1016/j.soilbio.2013.07.003 (2013).

15 Rousk, J., Smith, A. R. & Jones, D. L. Investigating the long-term legacy of drought and warming on the soil microbial community across five European shrubland ecosystems. *Global Change Biol* **19**, 3872-3884, doi:Doi 10.1111/Gcb.12338 (2013).

16 Li, Q. *et al.* Nitrogen Addition and Warming Independently Influence the Belowground Micro-Food Web in a Temperate Steppe. *Plos One* **8**, doi:DOI 10.1371/journal.pone.0060441 (2013).

17 Zelikova, T. J., Housman, D. C., Grote, E. E., Neher, D. A. & Belnap, J. Warming and increased precipitation frequency on the Colorado Plateau: implications for biological soil crusts and soil processes. *Plant Soil* **355**, 265-282, doi:DOI 10.1007/s11104-011-1097-z (2012).

18 Zavalloni, C. *et al.* Exposure to warming and CO2 enrichment promotes greater above-ground biomass, nitrogen, phosphorus and arbuscular mycorrhizal colonization in newly established grasslands. *Plant Soil* **359**, 121-136, doi:DOI 10.1007/s11104-012-1190-y (2012).

19 Yergeau, E. *et al.* Shifts in soil microorganisms in response to warming are consistent across a range of Antarctic environments. *Isme J* **6**, 692-702, doi:DOI 10.1038/ismej.2011.124 (2012).

20 Weedon, J. T. *et al.* Summer warming accelerates sub-arctic peatland nitrogen cycling without changing enzyme pools or microbial community structure. *Global Change Biol* **18**, 138-150, doi:DOI 10.1111/j.1365-2486.2011.02548.x (2012).

21 Song, B. *et al.* Light and Heavy Fractions of Soil Organic Matter in Response to Climate Warming and Increased Precipitation in a Temperate Steppe. *Plos One* **7**, doi:DOI 10.1371/journal.pone.0033217 (2012).

22 Shi, F. S., Chen, H., Chen, H. F., Wu, Y. & Wu, N. The combined effects of warming and drying suppress CO2 and N2O emission rates in an alpine meadow of the eastern Tibetan Plateau. *Ecol Res* **27**, 725-733, doi:DOI 10.1007/s11284-012-0950-8 (2012).

23 Long, X., Chen, C. R., Xu, Z. H., Linder, S. & He, J. Z. Abundance and community structure of ammonia oxidizing bacteria and archaea in a Sweden boreal forest soil under 19-year fertilization and 12-year warming. *J Soil Sediment* **12**, 1124-1133, doi:DOI 10.1007/s11368-012-0532-y (2012).

24 Yin, H. J., Xu, Z. F., Chen, Z., Wei, Y. Y. & Liu, Q. Nitrogen transformation in the rhizospheres of two subalpine coniferous species under experimental warming. *Appl Soil Ecol* **59**, 60-67, doi:DOI 10.1016/j.apsoil.2012.03.013 (2012).

25 Yin, H. J., Chen, Z. & Liu, Q. Effects of experimental warming on soil N transformations of two coniferous species, Eastern Tibetan Plateau, China. *Soil Biol Biochem* **50**, 77-84, doi:DOI 10.1016/j.soilbio.2012.03.004 (2012).

26 Kuffner, M. *et al.* Effects of season and experimental warming on the bacterial community in a temperate mountain forest soil assessed by 16S rRNA gene pyrosequencing. *Fems Microbiol Ecol* **82**, 551-562, doi:DOI 10.1111/j.1574-6941.2012.01420.x (2012).

27 Jassey, V. E. *et al.* Above‐and belowground linkages in Sphagnum peatland: climate warming affects plant‐microbial interactions. *Global Change Biol* **19**, 811-823 (2013).

28 Schindlbacher, A. *et al.* Experimental warming effects on the microbial community of a temperate mountain forest soil. *Soil Biol Biochem* **43**, 1417-1425, doi:DOI 10.1016/j.soilbio.2011.03.005 (2011).

29 Zhang, N. L., Xia, J. Y., Yu, X. J., Ma, K. P. & Wan, S. Q. Soil microbial community changes and their linkages with ecosystem carbon exchange under asymmetrically diurnal warming. *Soil Biol Biochem* **43**, 2053-2059, doi:DOI 10.1016/j.soilbio.2011.06.001 (2011).

30 Ma, L. N. *et al.* The Effects of Warming and Nitrogen Addition on Soil Nitrogen Cycling in a Temperate Grassland, Northeastern China. *Plos One* **6**, doi:DOI 10.1371/journal.pone.0027645 (2011).

31 Rinnan, R., Michelsen, A. & Baath, E. Fungi Benefit from Two Decades of Increased Nutrient Availability in Tundra Heath Soil. *Plos One* **8**, doi:DOI 10.1371/journal.pone.0056532 (2013).

32 Frey, S. D., Drijber, R., Smith, H. & Melillo, J. Microbial biomass, functional capacity, and community structure after 12 years of soil warming. *Soil Biol Biochem* **40**, 2904-2907, doi:DOI 10.1016/j.soilbio.2008.07.020 (2008).

33 Rinnan, R., Michelsen, A., Baath, E. & Jonasson, S. Fifteen years of climate change manipulations alter soil microbial communities in a subarctic heath ecosystem. *Global Change Biol* **13**, 28-39, doi:DOI 10.1111/j.1365-2486.2006.01263.x (2007).

34 Kandeler, E. *et al.* The response of soil microorganisms and roots to elevated CO2 and temperature in a terrestrial model ecosystem. *Plant Soil* **202**, 251-262, doi:Doi 10.1023/A:1004309623256 (1998).

35 Bardgett, R. D. *et al.* Below-ground microbial community development in a high temperature world. *Oikos* **85**, 193-203, doi:Doi 10.2307/3546486 (1999).

36 Gutknecht, J. L. M., Field, C. B. & Balser, T. C. Microbial communities and their responses to simulated global change fluctuate greatly over multiple years. *Global Change Biol* **18**, 2256-2269, doi:DOI 10.1111/j.1365-2486.2012.02686.x (2012).

37 A'Bear, A. D., Jones, T. H., Kandeler, E. & Boddy, L. Interactive effects of temperature and soil moisture on fungal-mediated wood decomposition and extracellular enzyme activity. *Soil Biol Biochem* **70**, 151-158, doi:DOI 10.1016/j.soilbio.2013.12.017 (2014).

38 Bergner, B., Johnstone, J. & Treseder, K. K. Experimental warming and burn severity alter soil CO2 flux and soil functional groups in a recently burned boreal forest. *Global Change Biol* **10**, 1996-2004, doi:DOI 10.1111/j.1365-2486.2004.00868.x (2004).

39 Nie, M. *et al.* Positive climate feedbacks of soil microbial communities in a semi-arid grassland. *Ecol Lett* **16**, 234-241, doi:Doi 10.1111/Ele.12034 (2013).

40 Zhang, X. M., Zhang, G. M., Chen, Q. S. & Han, X. G. Soil Bacterial Communities Respond to Climate Changes in a Temperate Steppe. *Plos One* **8**, doi:DOI 10.1371/journal.pone.0078616 (2013).

41 McDaniel, M. D., Kaye, J. P., Kaye, M. W. & Bruns, M. A. Climate change interactions affect soil carbon dioxide efflux and microbial functioning in a post-harvest forest. *Oecologia* **174**, 1437-1448, doi:DOI 10.1007/s00442-013-2845-y (2014).

42 Jumpponen, A. & Jones, K. L. Tallgrass prairie soil fungal communities are resilient to climate change. *Fungal Ecol* **10**, 44-57, doi:DOI 10.1016/j.funeco.2013.11.003 (2014).

43 Haugwitz, M. S. *et al.* Soil microorganisms respond to five years of climate change manipulations and elevated atmospheric CO2 in a temperate heath ecosystem. *Plant Soil* **374**, 211-222, doi:DOI 10.1007/s11104-013-1855-1 (2014).

44 Sun, X. F. *et al.* Diversity of arbuscular mycorrhizal fungal spore communities and its relations to plants under increased temperature and precipitation in a natural grassland. *Chinese Sci Bull* **58**, 4109-4119, doi:DOI 10.1007/s11434-013-5961-5 (2013).

45 Dennis, P. G. *et al.* Warming constrains bacterial community responses to nutrient inputs in a southern, but not northern, maritime Antarctic soil. *Soil Biol Biochem* **57**, 248-255, doi:DOI 10.1016/j.soilbio.2012.07.009 (2013).

46 Feng, X. J., Simpson, A. J., Wilson, K. P., Williams, D. D. & Simpson, M. J. Increased cuticular carbon sequestration and lignin oxidation in response to soil warming. *Nat Geosci* **1**, 836-839, doi:Doi 10.1038/Ngeo361 (2008).

47 Hart, S. C. Potential impacts of climate change on nitrogen transformations and greenhouse gas fluxes in forests: a soil transfer study. *Global Change Biol* **12**, 1032-1046, doi:DOI 10.1111/j.1365-2486.2006.01159.x (2006).

48 Clemmensen, K. E., Michelsen, A., Jonasson, S. & Shaver, G. R. Increased ectomycorrhizal fungal abundance after long-term fertilization and warming of two arctic tundra ecosystems. *New Phytol* **171**, 391-404, doi:DOI 10.1111/j.1469-8137.2006.01778.x (2006).

49 Olsrud, M. *et al.* Response of ericoid mycorrhizal colonization and functioning to global change factors. *New Phytol* **162**, 459-469, doi:DOI 10.1111/j.1469-8137.2004.01049.x (2004).

50 Rillig, M. C., Wright, S. F., Shaw, M. R. & Field, C. B. Artificial climate warming positively affects arbuscular mycorrhizae but decreases soil aggregate water stability in an annual grassland. *Oikos* **97**, 52-58, doi:DOI 10.1034/j.1600-0706.2002.970105.x (2002).

51 Ruess, L., Michelsen, A., Schmidt, I. K. & Jonasson, S. Simulated climate change affecting microorganisms, nematode density and biodiversity in subarctic soils. *Plant Soil* **212**, 63-73 (1999).

52 Hayden, H. L. *et al.* Changes in the microbial community structure of bacteria, archaea and fungi in response to elevated CO2 and warming in an Australian native grassland soil. *Environ Microbiol* **14**, 3081-3096, doi:DOI 10.1111/j.1462-2920.2012.02855.x (2012).

53 Zheng, Y. *et al.* Methanotrophic community structure and activity under warming and grazing of alpine meadow on the Tibetan Plateau. *Appl Microbiol Biot* **93**, 2193-2203, doi:DOI 10.1007/s00253-011-3535-5 (2012).

54 Zhang, W. *et al.* Soil microbial responses to experimental warming and clipping in a tallgrass prairie. *Global Change Biol* **11**, 266-277, doi:DOI 10.1111/j.1365-2486.2005.00902.x (2005).

55 Yoshitake, S. *et al.* Soil microbial response to experimental warming in cool temperate semi-natural grassland in Japan. *Ecol Res* **30**, 235-245 (2015).

56 Xu, Z., Zhao, C., Yin, H. & Liu, Q. Warming and forest management interactively affect the decomposition of subalpine forests on the eastern Tibetan Plateau: A four-year experiment. *Geoderma* **239**, 223-228 (2015).

57 van Meeteren, M. M., Tietema, A., van Loon, E. E. & Verstraten, J. M. Microbial dynamics and litter decomposition under a changed climate in a Dutch heathland. *Appl Soil Ecol* **38**, 119-127, doi:DOI 10.1016/j.apsoil.2007.09.006 (2008).

58 Jonasson, S., Michelsen, A., Schmidt, I. K. & Nielsen, E. V. Responses in microbes and plants to changed temperature, nutrient, and light regimes in the arctic. *Ecology* **80**, 1828-1843, doi:Doi 10.1890/0012-9658(1999)080[1828:Rimapt]2.0.Co;2 (1999).

59 Bradford, M. A. *et al.* Thermal adaptation of soil microbial respiration to elevated temperature. *Ecol Lett* **11**, 1316-1327, doi:DOI 10.1111/j.1461-0248.2008.01251.x (2008).

60 Xu, Z. *et al.* Initial responses of soil CO2 efflux and C, N pools to experimental warming in two contrasting forest ecosystems, Eastern Tibetan Plateau, China. *Plant Soil* **336**, 183-195, doi:10.1007/s11104-010-0461-8 (2010).

61 Andresen, L. C., Michelsen, A., Ambus, P. & Beier, C. Belowground heathland responses after 2 years of combined warming, elevated CO2 and summer drought. *Biogeochemistry* **101**, 27-42, doi:DOI 10.1007/s10533-010-9489-3 (2010).

62 Wang XJ, Zhou YM, Jiang XJ, & Han SJ. Effects of warming on soil microbial community structure in Changbai Mountain Tundra. *Acta Ecologica Sinica*, **20**, 5706-5713 (2014). (In Chinese with English abstract).

63 Wang B, Sun G, Luo P, Wang Z, Zhang Y, Wu N & Luo G. Microbial Communities of Alpine Meadow Soil in the Eastern Qinghai-Tibetan Plateau Subjected to Experimental Warming and Grazing. *Chin J Appl Environ Biol*, **17**, 151-157 (2011). (In Chinese with English abstract).

64 Wang XJ, Zhou YM, Wang XX, Jiang XJ, & Han SJ. Response of soil enzymes in activity and soil microbes in biomass to warming in tundra ecosystem on Changbai Mountains. *Acta pedologica sinica*, **51**, 166-175 (2014). (In Chinese with English abstract).
